# The Role of Cathepsins in Memory Functions and the Pathophysiology of Psychiatric Disorders

**DOI:** 10.3389/fpsyt.2020.00718

**Published:** 2020-07-24

**Authors:** Christine Niemeyer, Natalie Matosin, Dominic Kaul, Alexandra Philipsen, Nils C. Gassen

**Affiliations:** ^1^ Neurohomeostasis Research Group, Department of Psychiatry and Psychotherapy, University Hospital Bonn, Bonn, Germany; ^2^ Faculty of Science, Medicine and Health, Illawarra Health and Medical Research Institute, University of Wollongong, Wollongong, NSW, Australia; ^3^ Molecular Horizons, School of Chemistry and Molecular Biosciences, University of Wollongong, Wollongong, NSW, Australia; ^4^ Department of Translational Research in Psychiatry, Max Planck Institute of Psychiatry, Munich, Germany

**Keywords:** cathepsins, autophagy, memory function, neuronal plasticity, neuroinflammation

## Abstract

Cathepsins are proteases with functions in cellular homeostasis, lysosomal degradation and autophagy. Their role in the development of neurodegenerative diseases has been extensively studied. It is well established that impairment of proper cathepsin function plays a crucial role in the pathophysiology of neurodegenerative diseases, and in recent years a role for cathepsins in mental disorders has emerged given the involvement of cathepsins in memory function, hyperactivity, and in depression- and anxiety-like behavior. Here we review putative cathepsin functions with a special focus on their role in the pathophysiology of psychiatric diseases. Specifically, cathepsins are crucial for maintaining cellular homeostasis, particularly as part of the autophagy machinery of neural strategies underlying acute stress response. Disruption of cathepsin functions can lead to psychiatric diseases such as major depressive disease (MDD), bipolar disorder, and schizophrenia. Specifically, cathepsins can be excreted *via* a process called secretory autophagy. Thereby, they are able to regulate extracellular factors such as brain-derived neurotrophic factor and perlecan c-terminal fragment LG3 providing maintenance of neuronal homeostasis and mediating neuronal plasticity in response to acute stress or trauma. In addition, impairment of proper cathepsin function can result in impaired synaptic transmission by compromised recycling and biogenesis of synaptic vesicles. Taken together, further investigations on cathepsin functions and stress response, neuroplasticity, and synaptic transmission will be of great interest in understanding the pathophysiology of psychiatric disorders.

## Introduction

Cathepsins are molecular proteases found in all organisms. They are categorized into three groups according to their active site amino acid: cysteine cathepsins (B, C, F, H, K, L, O, S, V, W, and X), aspartic cathepsins (D and E), and serine cathepsins (G) (1). Apart from their protease activity, they are best known for their involvement in lysosomal degradation. In addition to these intracellular processes, extracellular activities of cathepsins have been recently described (2). Cathepsins are particularly involved in neuroregulation as many proteins produced by neurons are known substrates of cathepsins. The aim of this article is to highlight current research into cathepsin functions as well as their role in psychiatric disorders. Considering the current literature, we propose potential mechanisms of cathepsin involvement in the underlying pathophysiology of these conditions.

In the context of neurodegenerative diseases, such as Alzheimer’s disease (AD), it is well established that cathepsins are strongly implicated in disease progression (3). Since AD is characterized by hippocampal-dependent dysfunction, it is not surprising that cathepsins are also central to hippocampal-dependent learning and memory (4). Malfunction of memory performance is a common feature in most psychiatric disorders, making cathepsins an interesting target in understanding the pathophysiology underlying psychiatric diseases. Considering psychiatric illnesses account for a considerable amount of the global burden of disease, it is of great interest to identify altered genes and proteins that can be targeted by novel therapeutics to improve disease management. Autophagy is a process governed by cathepsins and also of emerging interest in the study of psychiatric conditions (5, 6). Classically, autophagy was considered as a non-selective, bulk degradation system. Recently, accumulating evidence revealed that autophagy can indeed selectively target proteins for degradation or can exert secretory functions *via* a process termed secretory autophagy (7). Thereby cathepsins can be secreted into the extracellular space with extracellular effects on neuroprotection and synaptic plasticity (2). To this end, a role for cathepsins in mental disorders is of emerging interest. In recent years, a number of studies have been published presenting cathepsins as crucial factors for maintaining proper memory function, as well as regulating hyperactivity, depression-, and anxiety-like behavior. However, the specific role of cathepsins in the development of psychiatric conditions is in its infancy.

## Cathepsins are Responsible for Hippocampus-Dependent Learning and Memory

Several studies in both humans and animals have demonstrated that cathepsins have fundamental functions related to learning and memory. For example, hippocampal cathepsin D (CTSD) tissue protein levels of *post mortem* brains were shown to display a quadratic relationship with cognitive function and episodic memory (8). That means, patients with *ante mortem* mild cognitive impairment have greater hippocampal CTSD levels compared to healthy controls and patients with AD (8). This finding could indicate that elevated CTSD levels characterize early stages of cognitive decline and function as a compensatory mechanism in response to mild neuronal damage.

Enhanced cathepsin B (CTSB) levels, on the other hand, were recently shown to improve cognition and memory function. Physical treadmill training over a four-week period was described to induce increased CTSB gene expression in the plasma of mice, monkeys, and humans (4). Its expression levels also correlated with hippocampus-dependent memory function (4). The same study provides evidence that CTSB crosses the blood–brain barrier and is capable of inducing the production of doublecortin and brain-derived neurotrophic factor (BDNF) in the brain (4), both important drivers of neuroplasticity (4, 9, 10).

However, an inverse correlation between both CTSB and BDNF protein levels, and weekly hours of exercise could be observed in a study with longer term physical training (>15 years) (11). In the study by de la Rosa and colleagues, the sample collection was performed at least 24 h after exercise, suggesting that physical exercise induces CTSB and BDNF acutely, but long-term adaptation results in lower expression, even compared to sedentary individuals (7).

In addition, experiments with mutant mice have demonstrated an involvement of cathepsin A (CTSA) and cathepsin K (CTSK) in spatial and non-spatial memory functions due to metabolic and structural changes in the hippocampus (12, 13). The catalytically inactive CTSA enzyme yields increased levels of oxytocin and endothelin-1. Both peptides are regulators of cellular pathways promoting long-term potentiation and spatial memory functions (14, 15). Specifically, oxytocin enhances long-term memory and long-term synaptic plasticity through the activation of the MAP kinase cascade and the consequent cyclic AMP-responsive element binding protein phosphorylation (15). Oxytocin and endothelin-1 are both substrates of CTSA, disruption of which yields accumulation of these peptides in the hippocampus (12), which is likely the cause for the deficits in learning and memory consolidation. CTSK is also highly active in the hippocampus. Its disruption has led to perturbated architecture of neuronal layers and decreased maturation of astrocytes (13). Consequently, a range of cathepsins are closely associated with proper hippocampus function. The hippocampus plays an important role in the pathophysiology of psychiatric disorders and abnormalities, e.g. atrophy, have repeatedly been observed in patients with major depressive disorder (16), posttraumatic stress disorder (17), and schizophrenia (18). Given their role in memory functions, cathepsins are likely involved in the pathophysiology of psychiatric disorders.

## Autophagy Processes are Orchestrated by Intracellular Cathepsins

Cathepsins are involved in cellular homeostasis by regulating apoptosis and autophagy. Different forms of autophagy (macroautophagy, microautophagy and chaperone-mediated autophagy) exist (19), but all pathways ultimately converge at the level of lysosomes, where engulfed biomolecules are degraded by cathepsins. Due to its role in protein degradation and recycling, autophagy is an essential process to ensure protein quality control and cellular homeostasis, and autophagy dysregulation has been implicated in a wide range of human diseases, including psychiatric disorders (5, 6). Constitutive autophagy is critical for neuronal survival. Mice with nervous system specific ablation of important autophagy genes (*Atg5* (autophagy-related 5) and *Atg7*) develop behavioral abnormalities like motor dysfunction and show cytoplasmatic inclusion bodies in the neurons (20, 21). Furthermore, recent studies point to a role of autophagy in the regulation of synaptic plasticity and neurotransmission by maintaining a counterbalance between protein synthesis and degradation (22), as well as maintaining the integrity of crucial organelles (e.g. synaptic vesicles) (23). Congruently, pharmacological upregulation of autophagy by rapamycin alleviated deficits in synaptic plasticity and improved cognitive impairments in male rats (24). The etiology and pathophysiology of psychiatric disorders is still very limited, but it was shown that impaired autophagy might contribute to the pathophysiology of psychiatric disorders, including schizophrenia (25), major depressive disorder (26), and bipolar disorder (27). Although the key factors are still unknown, it has been repeatedly proposed that disrupted synaptic plasticity mediated by impaired autophagy leads to the progression of psychiatric diseases. For example, autophagy dependent deregulation of activity-depended neuroprotective protein, an important factor for neuronal plasticity, was observed in *post mortem* hippocampus of schizophrenia patients (28). Congruently, mood stabilizers and antidepressants have been reported to affect autophagy. Specifically, lithium and antidepressants (amitriptyline, citalopram, paroxetine) were shown to enhance autophagy (29–32).

As summarized above cognition and psychiatric disorders exhibit characteristics of aberrated autophagy, which in turn results in impaired neuronal plasticity and protein aggregation, thereby contributing to disease pathophysiology. This process is likely to be partly orchestrated by cathepsins. Many cathepsins exhibit endopeptidase activity, i.e. they are capable of breaking peptide bonds of amino acids within the molecule, whereas some cathepsins exhibit exopeptidase activity, i.e. they are able to break peptide bonds of the end pieces of amino acids (1). If the function of cathepsins, and hence lysosomal function, is compromised, pathological conditions like inclusion body accumulation, impaired neurotransmission, and reduced neuroplasticity, can arise.

## Cathepsins in Psychiatric Disorders: Evidence From Patients, *Post Mortem* Brains and Animal Model Studies

In studying the pathophysiology of psychiatric disorders, cathepsins B, C, D and K are of particular interest. The following section provides an overview of the most intriguing findings of the involvement of these cathepsins in the pathophysiology of psychiatric disorders, in murine models, patients and *post mortem* brains.

### Anxiety- and Depressive-Like Disorders: Evidence From Murine Models

Transcriptome analysis of inbred mouse lines, selecting for low or high anxiety-related behavior with depression-like behavior, revealed higher CTSB levels in low anxiety mice (33). Accordingly, loss of CTSB expression resulted in an increase in anxiety-like and depression-like behavior (33). This effect, however, was only present in female mice. On the other hand, Zhanaeva and colleagues have demonstrated that when mice underwent chronic social defeat, a behavioral paradigm that leads to the development of a depressive-like phenotype in rodents, they demonstrate increased activity of CTSB in the hypothalamus and caudate nucleus (34). In line with these findings, paroxetine, a potent antidepressant, has been shown to downregulate CTSB protein abundance (35). A study describing a CTSK knockout mouse model revealed a reduction of anxiety-like behavior that was associated with an increase in dopamine and dopamine receptor D2 levels (13). This is in agreement with the finding that D2 receptor agonists induce learning impairments (36) and decrease anxiety levels in mice (37). Cathepsin C (CTSC) has also been suggested to play a critical role in the development of depression. In another study, CTSC overexpression and CTSC conditional knock-down mice were subjected to acute stress (lipopolysaccharide (LPS) injection) and chronic stress (6-week unpredictable chronic mild stress). Behavioral testing in these mice revealed that an overexpression of CTSC promoted the induction of depression-like behavior whilst CTSC knock-down was protective against this behavior (38). Depression-like behavior in CTSC overexpression was also shown to be associated with increased neuroinflammation and decreased 5-hydroxytryptamin (serotonin) levels (38). This finding is in accordance with the monoamine theory postulating decreased serotonergic neurotransmission as one of the prevailing causes for the development of depression (39).

In sum, studies with animal models revealed that deleterious CTSC and CTSK protected against anxiety- and depressive-like behavior. Furthermore, high levels of CTSB seem to have protective effects against these disorders in one study, while they promote depressive-like behavior in another. It will therefore be of great importance to further investigate the specific patterns of altered cathepsin expression among different cathepsins and across various brain areas to fully understand how cathepsins contribute to these disease entities.

### Bipolar Disorder: Evidence From Murine Models and Patients

In two distinct studies, CTSD knockout mice exhibited hyperactivity (40, 41), a hallmark of attention deficit hyperactivity disorder (ADHD) and mania. Behavioral testing of CTSD heterozygous knockout mice revealed a behavioral phenotype similar to human bipolar disorder (BD). This phenotype was comprised of increased general locomotor activity, decreased habituation, sleep disturbances as well as less anxious and more exploratory depression-like behavior (40). Furthermore, these behavioral features were sensitive to treatment with the mood stabilizers lithium and valproic acid (40), two classical therapeutics used in the management of BD.

Interestingly, in a study aiming for biomarkers to distinguish BD from ADHD it was shown that CTSB and CTSD gene expression is elevated in patients suffering from ADHD compared to BD patients (42), supporting that CTSD plays a central role in the pathophysiology of these disorders. However, it is important to note, that this study lacks information on CTSD levels of healthy subjects as control.

A genetic mouse model with deleterious CTSD resulted in a BD phenotype, while in patients high levels of CTSD is rather indicative for ADHD compared to BD. It is therefore likely that CTSD is downregulated in BD and ADHD, but to different extent.

### Autism Spectrum Disorder (ASD): Evidence From Murine Models and *Post Mortem* Brains

In an ASD murine model, elevated CTSB protein expression in neutrophils was observed (43). Given the role of neuroinflammation and microglia activation in the pathophysiology of ASD (44) this finding points to a mechanistic role of CTSB in inducing neurovascular inflammation. In addition, CTSD protein expression was found to be significantly increased in different regions of *post mortem* brains of ASD patients associated with increased apoptosis capacity (45). **Table 1** offers an overview of the most intriguing findings of cathepsins involved in the pathophysiology of psychiatric disorders found in *post mortem* brains.

**Table 1:** Findings on cathepsin involvement in psychiatric conditions from *post mortem* brains.

Target	Brain area	Disease (n)	Method	Finding	Ref.
CTSD	Brodmann Area 9	Schizophrenia and control (sample size not stated)	Enzyme assays	No significant differences in CTSD activity	(46)
CTSD	Anterior hippocampus	Schizophrenia (n = 7) vs. controls (n = 7)	2-D gel electrophoresis and mass spectrometry	Reduced CTSD protein expression in schizophrenia	(47)
CTSD	Mid hippocampus: cornu ammonis (CA) regions 1 through 4, dentate gyrus (DG)	Schizophrenia, bipolar disorder, and control subjects (n = 20 per group)	Laser-assisted microdissection to enrich for tissue from the hippocampal regions and 2-dimensional difference gel electrophoresis to compare protein profiles	Schizophrenia: DG and CA2/3 CTSD differentially expressed Bipolar disorder: no difference in CTSD expression level	(48)
CTSD	Prefrontal cortex Broadmann’s Area 10	Bipolar disorder, major depression, schizophrenia, and control subjects (n = 15 per group)	RNA microarray, real-time quantitative PCR	Upregulation of CTSD with aging; no between group comparison.	(49)
CTSD	Cerebellum, frontal cortex, hippocampus	Autistic subjects and age-matched control subjects (n = 7)	Immunhistochemistry, Western blots	Upregulation of CTSD expression in patients with autism in investigated brain areas	(45)
CTSK	Arcuate nucleus, paraventricular nucleus	Schizophrenia (n = 7) vs. control (n = 7)	Immunohistochemistry and morphometric analysis	Upregulation of CTSK expression in many brain regions of schizophrenic patients including the hypothalamus. Increase of the density of CTSK expressing hypothalamic and extrahypothalamic neurons in schizophrenia	(50)
CTSK	Cortex, Hypothalamus, supraoptic nucleus neurons and cerebellar Purkinje cells	Schizophrenia (n = 4) vs. controls (n = 4)	Immunohistochemistry, Western blots	Upregulation of CTSK expression in schizophrenia in investigated brain areas	(51)

### Schizophrenia: Evidence From Patients and *Post Mortem* Brains

In *post mortem* brains of patients with schizophrenia, CTSD protein abundance has been observed to be reduced in schizophrenia, compared to healthy controls (47). On the other hand, an upregulation of CTSK seems to be associated with schizophrenia. An analysis of *post mortem* brains of individuals with schizophrenia has identified an upregulation of CTSK expression, compared to matched controls (51). CTSK has been suggested to contribute to disease development by liberating enkephalin from beta endorphin (50). However, treatment with neuroleptics also upregulates CTSK in rat brain tissue (52). Therefore, it is difficult to differentiate whether the increase in CTSK expression is due to the long-term drug treatment rather than a result of the condition itself.

## Extracellular Cathepsins Maintain Neuronal Homeostasis and Induce Neuronal Plasticity

Although cathepsins are important proteases in the endolysosomal pathway, extracellular activity of these proteases is of emerging scientific interest (for a comprehensive review, see (2)). Cathepsins were shown to be involved in specific and non-specific degradation of proteins of the extracellular matrix (53). These findings point to a crucial role of cathepsins in shaping the (neuronal) microenvironment and maintaining tissue homeostasis. Therefore, attention has been drawn towards investigating their extracellular role as therapeutic and diagnostic targets. Recently, this has been seen within the context of neuroinflammation and axon growth. Extracellular cathepsin L (CTSL), for example, was shown to be capable of inducing axonal growth in neurons (54). Similarly, CTSB was found to degrade chondroitin sulfate proteoglycans, an inhibitor of axonal growth, thereby enhancing axonal outgrowth (54, 55). Furthermore, CTSL and CTSB elevate levels of the perlecan c-terminal fragment LG3, thereby mediating the beneficial effects of this fragment, including astrogliosis and neuroprotection (56). In addition, CTSD affects neuronal differentiation by processing members of the seizure-related gene family (SEZ6L2), which extracellularly exert differentiation (57).

**Figure 1:**
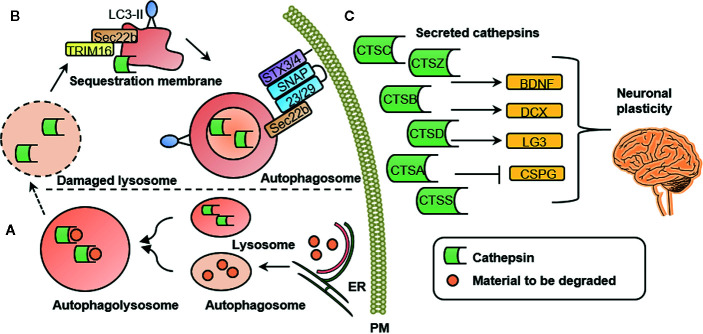
Cathepsin secretion, intracellular and extracellular cathepsin function. **(A)** Intracellular cathepsin function. Autophagosomes are generated at subdomains of the endoplasmic reticulum (ER). Upon closure of the membrane sac, the biomolecules to be degraded are enclosed in a double-membrane vesicle, called an autophagosome. Degradation is achieved by fusion with a lysosome to form an autophagolysosome. **(B)** Secretory autophagy pathway. Upon lysosomal damage, cathepsins are released into the cytosol. Cathepsins and other lysosomal proteins activate the galectin-8–TRIM 16 complex. TRIM16 binds to the cargo (here: cathepsins) to be excreted by secretory autophagy. A complex with TRIM16 and Sec22b is formed to transfer molecules (here: cathepsins) to the lipidated LC3 (often referred to as LC3-II) membrane. Sec22b then mediates fusion with the plasma membrane in conjunction with the SNARE molecules SNAP-23 and SNAP-29 as well as syntaxin 3 (STX3) and 4 (STX4). By this process, the cargo (here: cathepsins) is released into the extracellular space where it can exert their functions. **(C)** Extracellular cathepsin function. Once secreted into the extracellular space, cathepsins are involved in mediating neuronal plasticity. This is mediated through induction of BDNF, doublecourtin (DCX) and the perlecan c-terminal fragment LG3 (LG3) and by inhibition of chondroitin sulfate proteoglycans (CSPG). PM, plasma membrane; CTSS, cathepsin S; CTSZ, cathepsin Z (modified from (7), permission obtained from the authors).

It has been proposed that mental illness is caused by functional and structural changes in the brain. These are associated with axonal growth, neuroinflammation, and changes in cortical microglia, among a number of other effects (58). Changes in microglia markers have been reported in a number of psychiatric conditions including anxiety (59), depression (60, 61), and schizophrenia (62). Besides often reported cytokines, such as proinflammatory interleukin *β* (IL-1*β*), activated microglia excrete high levels of cathepsins (63). A secretome analysis of monocytes suggested that cathepsins A, B, C, D, S, and Z are secreted *via* a proteostatic process called secretory autophagy (7). **Figure 1** illustrates this highly specialized mechanism by which cathepsins are excreted into the extracellular space in more detail. Once secreted into the extracellular space, cathepsins are involved in mediating neuroplasticity through various processes. Cathepsins are capable of inducing BDNF (4), a well-known regulator of neuronal plasticity, synaptic plasticity, cell survival and differentiation, as well as doublecourtin, an important factor for neuronal migration (47).

## Conclusion and Outlook

Over the past few decades, a plethora of studies have been published investigating the pathophysiology and treatment of psychiatric disorders. Despite great progress, the underlying mechanisms still remain far from well-understood. Psychiatric disorders pose a particular challenge to research due to lack of reliable biomarkers and hence limitations to experimental design by relying on the individuals’ perception of their environment rather than objective measurements.

Disrupted cathepsin function or expression levels in animal models leads to behavioral phenotypes similar to BD, MDD, and anxiety like disorders in humans (13, 33, 34, 38, 40, 41). In *post mortem* brains of patients suffering from schizophrenia or ASD alterations of cathepsin levels have been found (45–51). The underlying molecular mechanisms of how cathepsin dysfunction contributes to the pathophysiology of psychiatric disorders remain unknown.

On a cellular level, cathepsins can influence axonal growth either directly *via* stimulation or indirectly *via* degradation of inhibitors (54–56). We deduce that these processes take place in response to acute stress or trauma to protect the brain as a short-term adaptation. However, not all cathepsins are protective for the brain. For example, overexpression of CTSC in the brain resulted in a depression-like phenotype (38). Furthermore, as observed from animal models lacking proper cathepsin function, the protective capabilities of cathepsins do not seem to be a simple cause and effect relationship. While short term cathepsin release could be beneficial, prolonged excretion might be harmful to the organism. During secretory autophagy, other factors such as high concentrations of chemokines, especially IL-1*β*, are also released (7). Prolonged secretion of IL-1*β* induces neuroinflammation thereby contributing to disease conditions. In agreement with this, CTSD was shown to trigger cytokine secretion (64) and cathepsin Z-deficient mice have significantly lower levels of IL-1*β* and reduced neuroinflammation (65). If cathepsins are highly excreted into the extracellular space, their intracellular concentration is reduced, thereby compromising their lysosomal capacity, i.e. the cell capacity to degrade molecules. This may lead to pathologies like neurodegenerative disorders (66) but also to psychiatric disorders (67). For example, in *post mortem* brain samples of schizophrenic patients and patients suffering from affective disorders protein aggregates of disrupted-in-Schizophrenia 1 (DISC1) (68) and dysbindin-1 (69) were found. The disposal of damaged proteins is essential for maintaining neuronal homeostasis, predominantly governed by autophagy and carried out by cathepsins. Congruently, in autophagy-compromised neurons protein accumulation was observed (20, 21). Suppressed autophagy also leads to reduced synaptic neurotransmission, while increased autophagy enhances transmission (70). This describes a central role for cathepsins in synaptic regulation. Thus, disruption of cathepsin signaling, such as CTSD, was shown to compromise the biogenesis of GABAergic synaptic vesicles and GABAergic synaptic transmission (41). Specifically, this led to reduced amplitudes of inhibitory postsynaptic currents, while excitatory postsynaptic currents were largely unaffected reflecting an imbalance between excitatory and inhibitory synaptic activity (41).

In light of the complex pathophysiology of psychiatric disorders, we are still far away from understanding the comprehensive mechanisms of cathepsins in these conditions. Induction of secretory autophagy and cathepsin-mediated neuroplasticity in response to acute stress are likely involved, but clarification is necessary before this can be confirmed. It will be of great interest to further investigate the conditions under which cathepsin expression and release into the extracellular space change and to further look into the effects of varying cathepsin levels on a cellular level, particularly in terms of synaptic neurotransmission. The diversity of roles for this class of proteases evidenced in the brain and throughout the body is important in the pathophysiology of a number of conditions and particularly of psychiatric disorders. Better understanding their effects within these systems may provide new avenues for better understanding how to combat some of the most complex human disorders.

## Author Contributions

CN drafted the manuscript. NM provided information on post mortem material. DK proofread the manuscript. NM, DK, AP, and NG added critical information. All authors contributed to the article and approved the submitted version.

## Conflict of Interest

The authors declare that the research was conducted in the absence of any commercial or financial relationships that could be construed as a potential conflict of interest.
